# Regulatory T cells regulate blastemal proliferation during zebrafish caudal fin regeneration

**DOI:** 10.3389/fimmu.2022.981000

**Published:** 2022-08-17

**Authors:** Subhra P. Hui, Kotaro Sugimoto, Delicia Z. Sheng, Kazu Kikuchi

**Affiliations:** ^1^ Developmental and Stem Cell Biology Division, Victor Chang Cardiac Research Institute, Darlinghurst, NSW, Australia; ^2^ S. N. Pradhan Centre for Neurosciences, University of Calcutta, Kolkata, West Bengal, India; ^3^ Department of Basic Pathology, Fukushima Medical University School of Medicine, Fukushima, Japan; ^4^ St. Vincent’s Clinical School, University of New South Wales, Kensington, NSW, Australia; ^5^ Department of Regenerative Medicine and Tissue Engineering, National Cerebral and Cardiovascular Center Research Institute, Suita, Japan

**Keywords:** Tregs, blastema, fin regeneration, growth factors, zebrafish

## Abstract

The role of T cells in appendage regeneration remains unclear. In this study, we revealed an important role for regulatory T cells (Tregs), a subset of T cells that regulate tolerance and tissue repair, in the epimorphic regeneration of zebrafish caudal fin tissue. Upon amputation, fin tissue-resident Tregs infiltrate into the blastema, a population of progenitor cells that produce new fin tissues. Conditional genetic ablation of Tregs attenuates blastemal cell proliferation during fin regeneration. Blastema-infiltrating Tregs upregulate the expression of *igf2a* and *igf2b*, and pharmacological activation of IGF signaling restores blastemal proliferation in Treg-ablated zebrafish. These findings further extend our understandings of Treg function in tissue regeneration and repair.

## Introduction

The ability to regenerate appendages after amputation varies greatly among species. Unlike mammals, fish and urodele amphibians fully restore lost appendages such as caudal fin and limbs at any stage of their lifetime (1–4). The restoration of appendages is mediated through epimorphic regeneration, which involves the formation of a cluster of undifferentiated cells, called the blastema, underneath the wound epidermis that covers the damaged tissue (1, 2, 5–7). During the appendage regeneration, blastemal cells vigorously proliferate and differentiate into all cell types that consist of appendage tissue, such as skin, bone, muscle, nerves, and blood vessels, and eventually restore the lost appendage tissue (7–11). Recent studies have identified molecular signals required for the blastemal cell proliferation (12–16). Inflammatory signals and innate immune cells such as macrophages have also been suggested to play critical roles in the appendage regeneration (17–22).

The caudal fin of zebrafish has been recognized as a standard model for appendage regeneration research (23, 24). Moreover, the zebrafish possesses an adaptive immune system comprising of T cells and B cells (25–30), which makes this model useful to study the role of adaptive immune cells during appendages regeneration. Although innate immune cells have been investigated in the zebrafish fin regeneration (31–35), the functional role of adaptive immune cells in epimorphic fin regeneration remains unclear. Using a transgenic cell ablation model and genetic mutants, here we have identified a novel role of Tregs in caudal fin regeneration, whereby Tregs promote blastemal cell proliferation by producing Igf2a and Igf2b in a fin-specific manner. This study extends our understanding of the non-immunological function of Tregs in tissue regeneration and repair.

## Materials and methods

### Zebrafish

Ekkwill (EK) or EK/AB mixed background zebrafish were outcrossed, and the both sexes were used in this study. The following published transgenic strains were used: *TgBAC(foxp3a:TagRFP)^vcc3^, TgBAC(lck:EGFP)^vcc4^*, *TgBAC(foxp3a:TagCFP-NTR)^vcc5^* (36, 37). All transgenic strains were analyzed as hemizygote conditions. The zebrafish carrying the *foxp3a^vcc6^* allele was described previously (36). During all experimental procedures, the fish density was maintained at 3–5 fish/L. The zebrafish husbandry and all experiments were performed according to the institutional and national animal ethics committee guidelines.

### Injury procedures

Zebrafish at 4-12 months of age were used for caudal fin amputation experiments. Caudal fin amputation was performed using a stereozoom microscope as described previously (2, 12). The amputation plane was set at 5 mm proximal from the cleft in the experiments for quantification of segmentation or at 2 mm proximal from the cleft in the other experiments.

### RT-PCR

Total RNA was extracted using TRIzol reagent, and cDNA was subsequently synthesized with SensiFAST™ cDNA Synthesis Kit (BIOLINE). qRT-PCR was performed using a LightCycler 480 system (Roche). For semi-qRT-PCR, genes of interest were amplified using a PrimeSTAR GXL kit (Clontech, Palo Alto, CA, USA). The amount of cDNA was normalized according to *actb2/β-actin2* amplification in qRT-PCR and semi-qRT-PCR experiments. The primers used in this study are listed in **Supplementary Table S1**.

### Antibodies

The following primary antibodies were used in this study: anti-active Caspase-3 (rabbit; Abcam, Cambridge, UK), anti-GFP (chicken; Abcam, Cambridge, UK), anti-tCFP (rabbit; Evrogen, Moscow, Russia), anti-tRFP (rabbit; Evrogen, Moscow, Russia), and anti-H3P (rabbit; Millipore, USA). The following secondary antibodies were used in this study: Alexa Fluor 488 donkey anti-rabbit IgG(H+L), Alexa Fluor 488 goat anti-chicken IgY(H+L), and Alexa Fluor 555 donkey anti-rabbit IgG(H+L) (Life Technologies, USA).

### Imaging

Wholemount images of caudal fins were taken by a stereo-fluorescence microscope (Olympus, Japan). Fish were anesthetized by tricaine and were laid on a petri dish filled with aquarium water, followed by fluorescent exposure. For time-lapse imaging, images were manually taken every 20 min. Sample drift was corrected manually on Photoshop CS5 (Adobe) utilizing pigments as guides. EdU was detected using either Click-iT EdU Alexa 488 or Alexa 555 Imaging kit (Life Technologies, USA). The fluorescence-stained samples were imaged using either a Zeiss AXIO imager M1 microscope (Carl Zeiss AG, Oberkochen, Germany) or a Zeiss LSM 710 confocal microscope (Carl Zeiss AG).

### *In situ* hybridization

*In situ* hybridization in fin tissue sections was performed using RNAscope probes (Advanced Cell Diagnostics, Hayward, CA). Regenerated fin tissues were fixed with 4% paraformaldehyde for 24 hours at 4°C and equilibrated in 30% sucrose for another 24 hours, embedded in a Tissue freezing medium (TFM; Leica Biosystems, Wetzlar, Germany), and cryosectioned to 10 µm. Fin sections were washed twice with PBS for 5 min to remove TFM, followed by incubation in hydrogen peroxide for 10 min at room temperature, boiling in target retrieval for 5 min. After target retrieval, slides were briefly washed with distilled water and incubated for 5 min at 40°C with Protease Plus. After the pretreatments in fin sections, the manufacturer’s protocol for RNAscope 2.5 HD Detection Kit-Red (Advanced Cell Diagnostics) was followed to hybridize the *igf2a* probe and detect the signals. Immunostaining using an anti-tRFP antibody was performed following the detection of *igf2a* mRNA signals. After the RNAscope assay and fin sections were incubated with primary antibody anti-tRFP overnight at 4°C. The *igf2a* RNA probe used in this study was designed and synthesized by Advanced Cell Diagnostics.

### Drug administrations

For Treg cell ablation experiments, *foxp3a:NTR* fish were placed in a small beaker of aquarium water containing 0.2% dimethyl sulphoxide (DMSO) and 15 mM freshly dissolved Mtz (M1547; Sigma, St. Louis, MO, USA). Fish were maintained in the dark and in this media for 10-12 hours (overnight), rinsed with fresh aquarium water, and returned to a recirculating aquatic system. For regeneration experiments, this treatment cycle was repeated for three consecutive days before fin amputation and afterward continued every other day until the collection of regenerated fins (**Figure 2A**). For the IGF-activator NBI-31772 treatment, the fish were placed in a small beaker filled with 30 ml of aquarium water containing 10 µl DMSO as a negative control or the same amount of 30 mM NBI-31772 dissolved in DMSO (final conc. 10 µM) for overnight. For the cell proliferation assay, adult zebrafish were intraperitoneally injected with 50 µl of 10 mM EdU once 30 min before the collection of regenerated fin tissues.

### Flow cytometry and cell sorting

To prepare caudal fin cell suspension, the fin blastema was dissected, and placed into a microcentrifuge tube containing 0.9× PBS with 1 mg/ml collagenase type 2 (Worthington Biochemical, Lakewood, NJ, USA), and incubated for 30 min at room temperature with gentle pipetting every 10 min. Dissociated cells were washed and re-suspended in ice-cold staining buffer. To prepare the cell suspensions from the kidney, spinal cord, and retina, the tissues were dissected in ice-cold 0.9× PBS with 5% fetal bovine serum (staining buffer) and placed on a cell strainer (40 µm; Falcon 2340). Next, the pool of individual soft tissues was pushed through the strainer with a syringe plunger. To prepare a cardiac cell suspension, the ventricle was dissected, placed into a microcentrifuge tube containing 0.9× PBS with 1 mg/ml collagenase type 2 (Worthington Biochemical, Lakewood, NJ, USA), and incubated for 40 min at room temperature with gentle pipetting every 10 min. Dissociated cells were washed and re-suspended in ice-cold staining buffer. Peripheral blood was obtained by puncturing the heart of the caudal fin of amputated fish. The collected blood was treated with ACK red blood cell lysing buffer (Gibco, Grand Island, NY, USA) and suspended in ice-cold staining buffer. FACS analysis was performed on a LSRII SORP (BD Biosciences, San Jose, CA, USA), and cell sorting was performed on a FACSAria IIu (BD Biosciences). Data were analyzed using FlowJo software (Treestar, Ashland, OR, USA). Dead cells, defined as those positively stained with DAPI (4’,6-diamidino-2-phenylindole), and doublet cells were excluded from all analyses and sorting. Cells in the lymphoid fraction were sorted in two sequential steps and collected directly during the second sort into a microcentrifuge tube containing 1 ml of TRIZOL reagent (Invitrogen) for subsequent RT-PCR analysis.

### Quantification of cells from microscopic images

All images for quantification were taken using a Zeiss AXIO imager M1 microscope with a 10× objective (Carl Zeiss AG). Treg cells were quantified in *foxp3a:RFP* fish by taking red fluorescence images of the regenerated caudal fin blastema areas (1388 × 278 pixels) and manually counting RFP^+^ cells using ImageJ software (US National Institutes of Health, Bethesda, MD, USA). The results from six selected sections were averaged to determine the number of RFP^+^ cells in each caudal fin.

To quantify proliferating blastemal cells, images of the fin blastema were taken at 4 dpa using Zeiss AXIO imager M1 microscope and areas (1024 × 1024 pixels). The numbers of either EdU^+^ and H3P^+^EdU^+^ cells were manually counted using ImageJ software. The number of H3PSox2^+^PCNA^+^EdU^+^ cells from twelve sections was analyzed to determine the number of proliferating blastemal cells in each regeneration time point. To quantify caudal fin regeneration length, images of the amputated fin, including the proximal area, were taken at 1, 2, 3, 4, 5, 7, and 10 dpa (1600 × 1200 pixels), and the length of regenerated blastema from the fin amputation plane was manually measured using ImageJ software. Fin blastema cells undergoing apoptosis were quantified as described above, except that the numbers of Edu^+^Caspase-3^+^ cells were counted in amputated fins.

## Results

**Figure 1:**
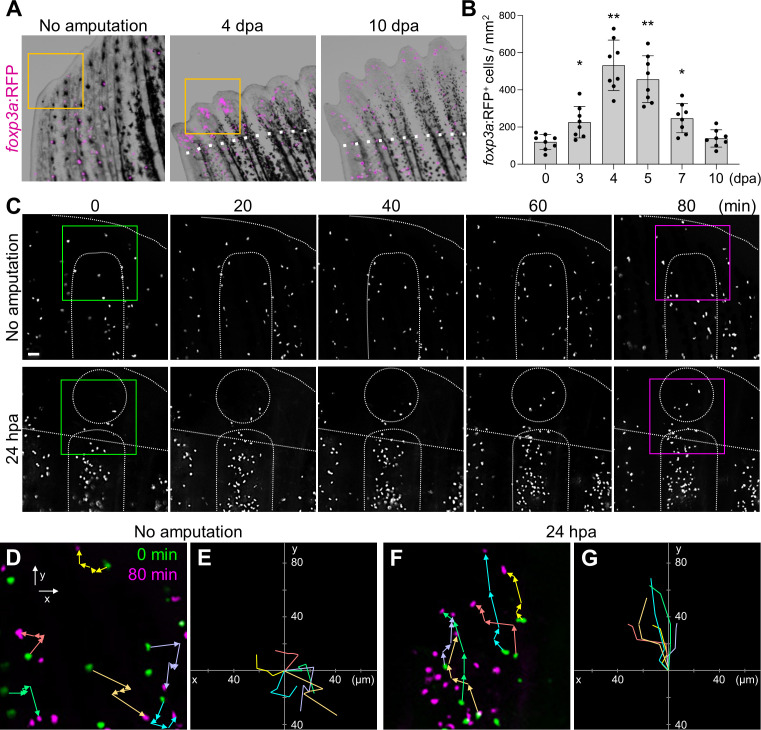
Amputation-induced infiltration of Tregs in the regenerating caudal fin tissue. **(A)** Spatio-temporal distribution of *foxp3a:*RFP+ Treg cells in the distal part of unamputated, 4 and 10 days post amputation (dpa) regenerating caudal fins. Dotted lines show the plane of amputation. Yellow box indicates the distal tip of unamputated fin or regenerating fin blastema. Bar, 200 µm. **(B)** Quantification of *foxp3a:*RFP+ cells in unamputated, 3, 4, 5, 7 and 10 dpa fins (mean ± SEM, n = 8, *P < 0.01, **P < 0.001, Mann–Whitney *U* test). **(C)** Time-lapse images of *foxp3a:*RFP+ cells in unamputated or 24 hours after amputation (hpa) of caudal fin. Dotted lines demarcate the distal tip, the bone, the blastema, and amputation planes. Bar, 20 µm. **(D, F)** Higher magnification fields of C in unamputated and 24 hpa fin respectively. *foxp3a:*RFP+ cells at 0 min and 80 min are indicated by green and magenta, respectively. Colored arrows correspond to the migratory tracks of each *foxp3a:*RFP+ cells in unamputated **(D)** and 24 hpa fin **(F)**. Bar, 20 µm. **(E, G)** Combinatorial overlay of the 6 individual tracks of *foxp3a:*RFP+ cell in **(D, F)**, respectively, and which were plotted after aligning their starting positions. Each crawling tracks display a migratory path for individual Treg cells. The traces shown in **(D, F)** are repositioned to the center to define the migratory path distance of each dot. Bar, 20 µm.

In order to visualize Tregs during caudal fin regeneration, we used a Treg-specific reporter line, *TgBAC(foxp3a:TagRFP)^vcc3^* (hereafter *foxp3a: RFP*), in which the expression of red fluorescent protein (TagRFP) is controlled by bacterial artificial chromosome containing *forkhead box P3a* (*foxp3a*) gene (37). A few *foxp3a*: RFP^+^ cells were found in the unamputated caudal fin (**Figure 1A**). After amputation of the caudal fin, the number of *foxp3a*:RFP^+^ cells was increased in the regenerating fin tissue, which peaked at 4 days post-amputation (dpa) and decreased to an uninjured fin level at 10 dpa (**Figures 1A, B**). Reverse transcriptase-quantitative polymerase chain reaction (RT-qPCR) analysis showed a similar temporal pattern of *foxp3a* expression in regenerating fin tissues (**Supplementary Figure S1A**).

Tregs were shown to mobilize to damaged tissues through the bloodstream during the regeneration of the heart, spinal cord, or retina in zebrafish (37). However, we did not detect a significant increase of *foxp3a*:RFP^+^ cells in peripheral blood after fin amputation (**Supplementary Figure S1B**), suggesting that Tregs unlikely mobilize to the regenerating fin through the bloodstream. To examine whether fin-resident Tregs contribute to fin regeneration, we performed time-lapse imaging of *foxp3a:RFP* fin after amputation (**Figures 1C−G**). In the uninjured fin, *foxp3a*:RFP^+^ cells did not show any evidence of directional migration during the period of imaging (**Figures 1C, D**). In contrast, in the injured fin, *foxp3a*:RFP^+^ cells were increased in the regenerating fin tissue (**Figure 1C**) with clear migratory paths directed to the amputation plane (**Figure 1D**). These data suggest that fin-resident Treg cells respond to injury and migrate to the site of regeneration of damaged fin tissues.

**Figure 2:**
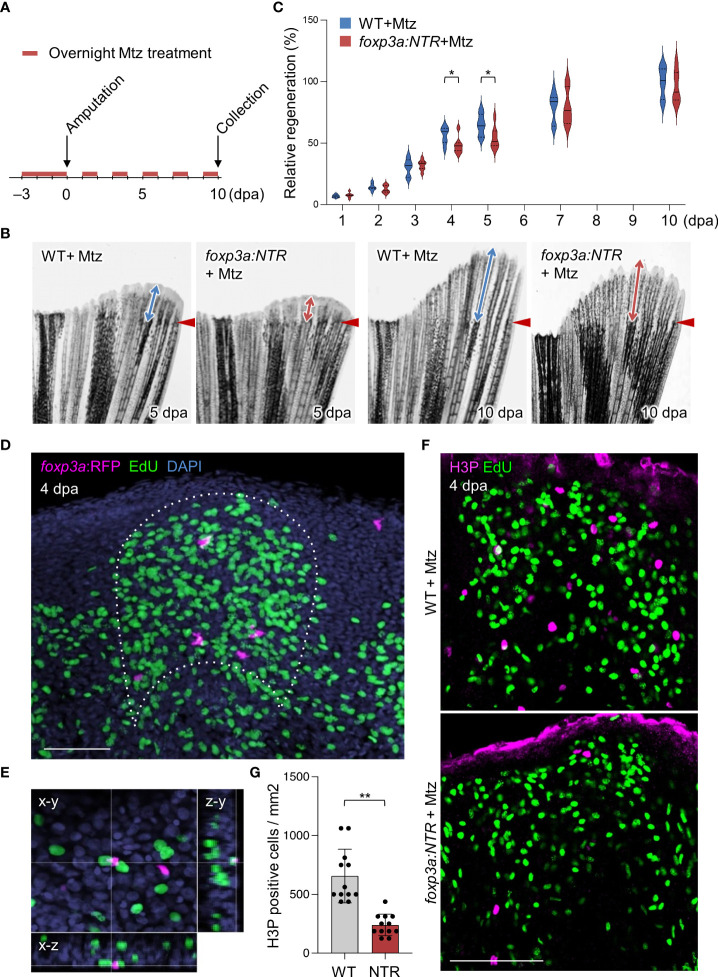
Tregs are required for blastemal proliferation during caudal fin regeneration. **(A)** Experimental scheme for Mtz application in *foxp3a:NTR* fish to achieve Treg cell-specific ablation and study caudal fin regeneration. Three continuous days of overnight treatment of Mtz were performed before the initiation of caudal fin amputation at day 0. **(B)** Brightfield microscopic images of caudal fins show the rate of fin regeneration after 5 and 10 dpa in wild-type and Treg cell ablated fish. **(C)** Rate of fin regeneration length was quantified in the wild-type fish against the Treg ablated fish. The average length of wild-type 10 dpa was considered as 100% length of fin regeneration (mean ± SEM, n = 7, Student’s T-test). **(D, E)** Confocal images of fin blastema at 4 dpa after EdU labeling indicates the *foxp3a:*RFP+ cells are spatially localized in close proximity of EdU+ blastemal cells **(D)** and sometimes they are also directly in contact with the EdU+ blastemal cells **(E)**. EdU was injected intraperitoneally 30 mins before the collection of fin tissue. **(F)** The wholemount preparation of 4 dpa fin with EdU and H3P immunostaining in wild-type and after Treg cell ablation. **(G)** Quantification of H3P+ cells in the 4 dpa fin blastema of wild type and Treg ablated fish (mean ± SEM, n = 12, Mann–Whitney *U* test). *P < 0.01; **P < 0.001; Mtz, metronidazole; NTR, nitroreductase; Scale Bars, 50 mm.

We next examined the function of Tregs during fin regeneration using the transgenic line *TgBAC(foxp3a:TagCFP-NTR)^vcc5^* (hereafter *foxp3a:NTR*) (37), in which a fusion protein of bacterial nitroreductase (NTR) and cyan fluorescent protein (TagCFP) is specifically expressed in Tregs. The NTR converts the pro-drug metronidazole (Mtz) to a cytotoxic agent in eukaryotic cells (38), and thus Tregs can be conditionally ablated in *foxp3a:NTR* fish with Mtz administrations. After the establishment of the fin-Treg depletion protocol (**Supplementary Figures S2A−G**), we analyzed the effect of Treg depletion on fin regeneration. We found a significant decrease in the outgrowth of regenerated fin tissue after Treg depletion (**Figures 2B, C**). But continuous observation of regenerated fin tissue growth until 10 dpa after Treg cells depletion revealed no obvious difference between WT and *foxp3a:NTR* fish (**Figure 2C**).

One of the most critical events in zebrafish fin regeneration is the proliferation of blastemal cells and which occurs during the outgrowth of fin tissue (1, 39, 40). The proliferating blastemal cells in the regenerating fin blastema can be detected by EdU labeling after fin amputation of EdU injected *foxp3a:RFP* fish. Interestingly, we observed Tregs in the blastema of 4 dpa fin are localized in close vicinity of EdU+ cells (**Figure 2D**), and sometimes they are associated directly with the EdU+ blastemal cells (**Figure 2E**), suggesting Tregs may have a role in blastemal cell proliferation. Thus to find out the basis behind the aberrant/reduced growth of regenerated fin after ablation of Treg cells, we looked at the rate of blastemal cell proliferation by H3P (Phospho-histone H3) immunostaining in EdU injected *foxp3a:NTR* fish. We found a significant reduction of blastemal cell proliferation at 4 dpa fin blastema after depletion of Treg cells compared to Mtz treated WT as measured by quantification of H3P+ cells (**Figures 2F**, G). Whereas, at the same time point (4 dpa) the number of dying/apoptotic cells and the expression of pro-survival genes in the blastema tissue remained unchanged after Treg cells depletion (**Supplementary Figures S2E−G**). Taken together, these data suggest Tregs are the essential regulator of fin regeneration, at least for the early stages, by promoting the proliferation of blastemal cells.

**Figure 3:**
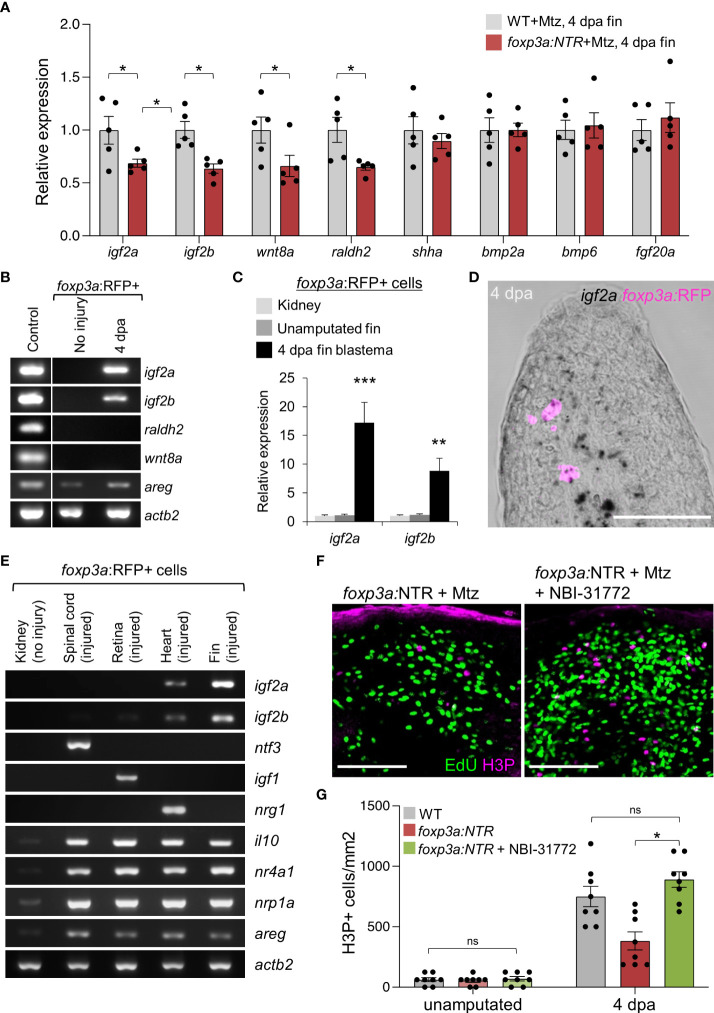
Blastemal cell proliferation during caudal fin regeneration is regulated by Treg cells-derived pro-regenerative factors. **(A)** qRT-PCR analysis of growth factors expression in 4 dpa fin blastema of wild-type and *foxp3a:NTR* fish after Mtz treatment. (mean ± SEM, n = 5, Student’s T-test). **(B)** RT-PCR analysis of growth factors expression (found significant decrease after Treg cell ablation) from purified *foxp3a:*RFP+ cells from unamputated and 4 dpa fin blastema. The 4 dpa fin blastema tissue was used as control. **(C)** Expression analysis of *igf2a* and *igf2b* in purified *foxp3a:*RFP+ cells from kidney marrow, unamputated fin and 4 dpa fin blastema. (mean ± SEM, n = 5, Student’s T-test). **(D)** *In situ* hybridization using RNAscope and immunofluorescence against TagRFP showing *igf2a* mRNA expression within infiltrated *foxp3a:*RFP+ cells of a 4 dpa fin blastema. **(E)** RT-PCR analysis of growth factor expression of purified *foxp3a:*RFP+ cells from kidney, 7 days post injured (dpi) spinal cord, 4 dpi retina, 7 dpi heart, and 4 dpa fin blastema tissues show tissue-specific growth factor secretion pattern of Treg cells. **(F)** The wholemount preparation of 4 dpa fin with EdU and H3P immunostaining in Treg ablated fish and fish with NBI-31772 application after Treg ablation. EdU was injected intraperitoneally at 30 min before fin tissue collection. **(G)** Quantification of H3P+ cells in unamputated and 4 dpa fin blastema of wild-type, Treg ablated fish, and fish with NBI-31772 application after Treg ablation (mean ± SEM, n = 8-9, Mann–Whitney *U* test). *P < 0.01; **P < 0.001; ***P < 0.0001; ns, not significant; Mtz, metronidazole; NTR, nitroreductase; Sacle Bars, 50 mm.

Tregs are increasingly known to regulate tissue repair and regeneration by providing growth factors in damaged tissues (37, 41, 42). Thus, to explore the mechanism behind the impaired blastemal cell proliferation in the absence of Tregs, we looked at the expression of known growth factors/signaling molecules directly influencing blastemal cell proliferation (12–16, 32, 43) after depletion of Treg cells. We have found that Treg cell depletion in 4 days of regenerated fin specifically reduced the expression of *igf2a, igf2b, wnt8a, and raldh2* (**Figure 3A**). To further investigate whether Tregs are the direct source of these growth factors, we performed gene expression analysis of particular growth factors from purified *foxp3a*:RFP^+^ cells from 4 dpa fin blastema and the unamputated fin (**Figure 3B**). The Treg cell-specific expression of *igf2a* and *igf2b* was detected by qRT-PCR analysis, and among them, *igf2a* was predominantly expressed from the regenerated fin-derived Tregs (**Figure 3C**). To confirm the *igf2a* expression from fin-derived Tregs, we performed a high-resolution *in situ* hybridization by RNAscope assay and detected the *igf2a* expressing *foxp3a*:RFP^+^ cells at the blastemal tissue of 4 dpa fin (**Figure 3D**).

To confirm whether the organ-specific secretary phenotype of Tregs also persists during zebrafish fin regeneration akin to our previous findings (37, 44, 45), we compared the *igf2a* and *igf2b* expression of fin blastema-derived Tregs with the injured spinal cord, retina, and heart derived Tregs. Strikingly, the gene expression of the pro-regenerative factors for the spinal cord (*ntf3*), the retina (*igf1*), and the heart (*nrg1*) was not detectable in the Tregs purified from fin blastema (**Figure 3E**). However, the expression of *igf2a* and *igf2b* were highly enriched in the fin blastema-derived Tregs (**Figure 3E**), suggesting that Tregs produce Igf2a and Igf2b to regulate blastemal proliferation. To examine the role of Tregs-derived Igf2a and Igf2b in blastemal proliferation, we exogenously administered the IGF signaling activator, NBI-31772, in Treg cells-ablated 4 dpa fin. The H3P^+^ blastemal cells were significantly increased in Treg cells-ablated 4 dpa fin after the treatment of fish with NBI-31772 compared to DMSO treatment, and the blastemal cell proliferation was restored similar to WT level (**Figures 3F, G**). Together, our results indicate that Tregs acquire a specific secretory phenotype in the regenerating fin tissue that activates IGF signaling to promote blastemal proliferation.

To determine whether the expression of *igf2a* and *igf2b* are regulated by the transcription factor Foxp3a, we used an established zebrafish mutant line, *foxp3a^vcc6^* (hereafter, *foxp3a^−/−^*) (36). We found that the expression of *igf2a* and *igf2b* was not detectable in Tregs purified from the injured *foxp3a^−/−^* fin tissues (**Supplementary Figure S3F**), indicating that the expression of *igf2a* and *igf2b* are directly regulated by transcription factor *foxp3a*. The lack of *igf2a* and *igf2b* expression resulted in the significant blastemal proliferation and fin growth in *foxp3a^−/−^* fish irrespective of a similar number of infiltrations of Tregs in 4 dpa fin blastema compared to WT (**Supplementary Figures S3A−E**). Moreover, as expected, the expression of pro-inflammatory genes *tnfa*, *ifng1-1*, and *il6* were also elevated in regenerating fin tissue of *foxp3a^−/−^* fish (**Supplementary Figure S3G**) compared to wild-type. These findings are consistent with our previous observation (37) that the secretion of pro-regenerative factors from Tregs is controlled by the transcription factor Foxp3a.

## Discussion

The formation of the blastema is a characteristic feature of epimorphic regeneration (2, 3, 46). In this study, we found that zebrafish Tregs regulate epimorphic fin regeneration by promoting blastemal cell proliferation. Consistent with our previous finding during spinal cord, retina, and heart regeneration in zebrafish, Tregs are also accumulated during fin regeneration. However, unlike those accumulated in the damaged spinal cord, retina, and heart, Tregs in the regenerating fin tissue are not mobilized through circulation but from the skin tissue nearby the fin amputation plane (**Figure 1C**). Future study is necessary to determine whether this is a unique mechanism for fin regeneration or whether the skin is a reservoir of Tregs that mobilize to damaged tissues in zebrafish.

Tregs accumulated in the injured fin tissue produce a unique set of pro-regenerative factors, Igf2a and Igf2b, to promote blastemal proliferation. In mammals, Tregs are also known to accumulate in the damaged skin tissue and promote repair by producing paracrine factors including Areg (41, 47–51). Tregs may have an ancestral and universal role in producing pro-regenerative factors in the response to tissue damage, and this mechanism could be targeted to enhance regeneration and repair of damaged tissues in humans.

The mechanism by which zebrafish Tregs acquire a tissue-specific secretory phenotype during regeneration remains unclear. Further studies with single-cell transcriptomics and epigenomics might explain the detailed molecular mechanism by which zebrafish Tregs modulate the tissue-specific expression of trophic and mitogenic factors in response to local injury niche. Decoding the molecular mechanism by which zebrafish Tregs acquire a tissue-specific pro-regenerative function may provide novel implications for future regenerative therapies targeting human Tregs.

## Data availability statement

The original contributions presented in the study are included in the article/**Supplementary Material**. Further inquiries can be directed to the corresponding authors.

## Ethics statement

The animal study was reviewed and approved by Victor Chang Cardiac Research Institute, Darlinghurst NSW 2010, Australia.

## Author contributions

Conceptualization, SH, KS, and KK. Methodology, KS, SH, DS, and KK. Investigation, KS, SH, DS, and KK. Writing – original draft, SH and KK. Writing – review and editing, SH, KS, and KK. Supervision, KK. Project administration, KS, SH, and KK. Funding acquisition, KK. All authors contributed to the article and approved the submitted version.

## Funding

This work is supported by grants from NHMRC (APP1130247) and JDRF (3-SRA-2018-604-M-B) to KK.

## Acknowledgments

We thank M. Nakayama and D. Zhang for technical assistance. C. Jenkin, J. Martin, and K. Brennan for zebrafish care. E. Lam and R. Salomon for flow cytometry and FACS. K. Kawakami for plasmids. and Sukla Ghosh for discussions and comments on the manuscript.

## Conflict of interest

The authors declare that the research was conducted in the absence of any commercial or financial relationships that could be construed as a potential conflict of interest.

## Publisher’s note

All claims expressed in this article are solely those of the authors and do not necessarily represent those of their affiliated organizations, or those of the publisher, the editors and the reviewers. Any product that may be evaluated in this article, or claim that may be made by its manufacturer, is not guaranteed or endorsed by the publisher.
