# Supplementary material for: Regulatory T cells regulate blastemal proliferation during zebrafish caudal fin regeneration
Source: Front Immunol. 2022 Aug 17;13:981000. doi: 10.3389/fimmu.2022.981000 (PMC9429828; doi:10.3389/fimmu.2022.981000)
Supplement: Supplementary file 1 [file DataSheet_1.pdf]

SUPPLEMENTAL INFORMATIONS

Figure S1–S3

Table S1

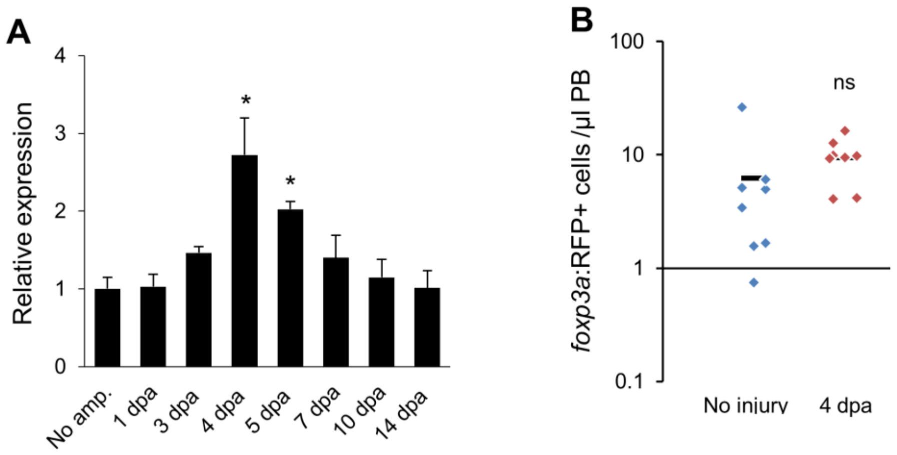

Supplementary Figure S1. Infiltration of Tregs during caudal fin regeneration.

(A) qRT-PCR of *foxp3a* was done from unamputated and regenerated fin blastema tissues from different time points (mean ± SEM, n = 5, *P < 0.01, Student's T-test). Unamputated fin tissue was used as control. (B) Quantification of sorted RFP+ cells from the peripheral blood (PB) of *foxp3a:RFP* fish without fin amputation and 4 days after fin amputation (4 dpa). (n = 8, ns = not significant, Mann–Whitney *U* test).

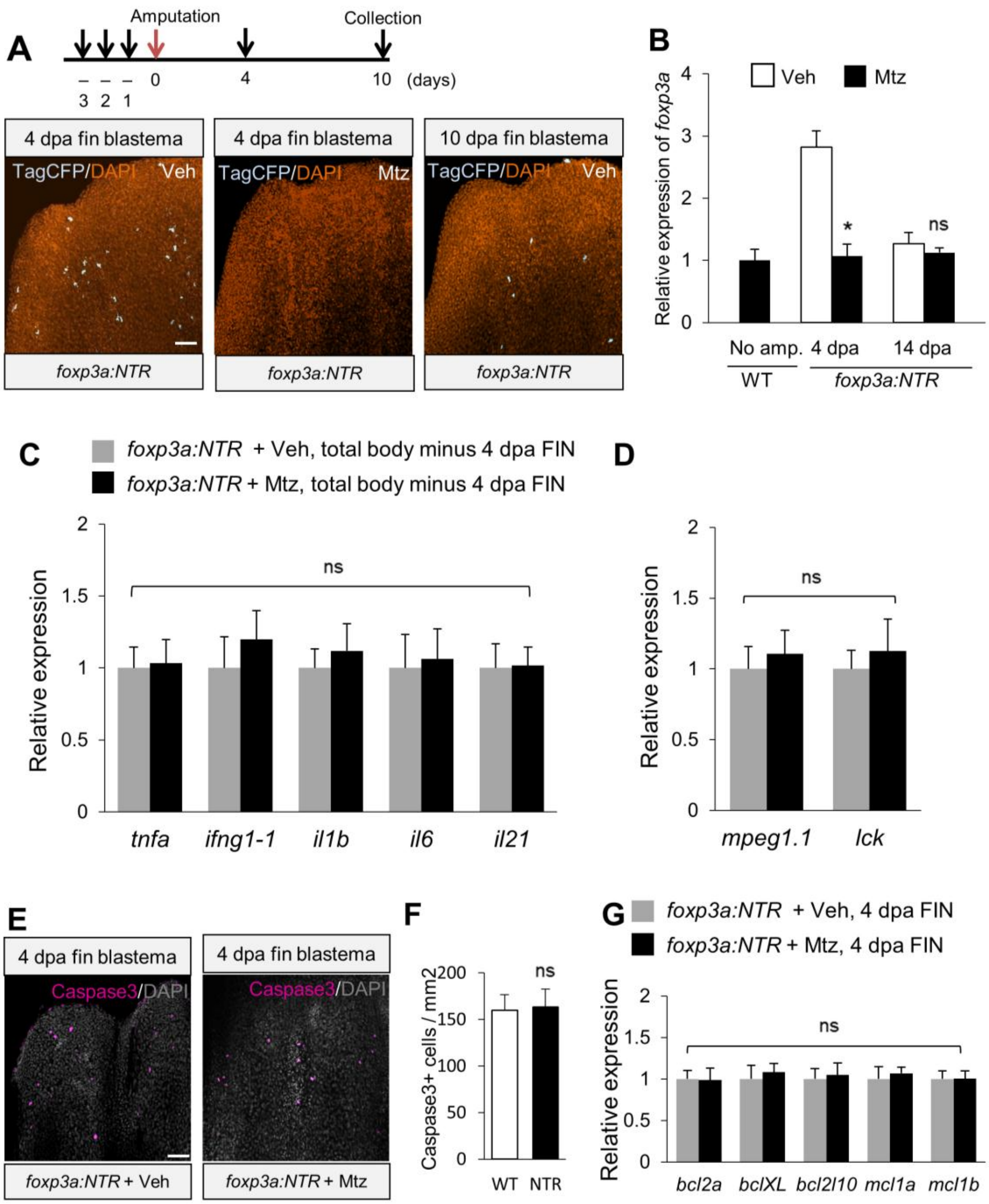

Supplementary Figure S2. Inducible ablation of Treg cells during caudal fin regeneration.

(A) Scheme for Treg cell ablation in which *foxp3a:NTR* fish were amputated at the distal region of caudal fin after 3 continuous overnight treatment with Veh or Mtz. The caudal fin blastema were analyzed via immunofluorescence against TagCFP and DAPI at 4 dpa and 10 dpa. (B) *foxp3a:NTR* fish were subjected to amputation and Veh or Mtz treatments

as described in scheme A and qRT-PCR of *foxp3a* were done from regenerated caudal fin tissue (mean ± SEM, n = 5, *P < 0.01, Student's T-test). Unamputated fin tissue was used as control. (C, D) *foxp3a:NTR* fish underwent 3 overnight treatments with vehicle (Veh) or metronidazole (Mtz), and total fish samples were used for qRT-PCR analysis (mean ± SEM, n = 5, Student's T-test) of inflammatory genes (C), macrophages and T cell marker (D). Gene expression is shown relative to the levels in vehicle controls. (E) Immunofluorescence against active Caspase-3. Images demonstrate the apoptotic blastemal cells in the 4 dpa fin blastema of *foxp3a:NTR* fish after treatment with Veh or Mtz. (F) Quantification of active Caspase-3+ cells in the 4 dpa fin blastema of *foxp3a:NTR* fish after treatment with Veh or Mtz (mean ± SEM, n = 5, Mann–Whitney *U*-test). (G) qRT-PCR analysis of cell survival factor gene expression in 4dpa blastemal tissues from wild type and Treg cell ablated fish (mean ± SEM, n = 5, Student's T-test). Mtz = Metronidazole, NTR = Nitroreductase, CFP = Cyan Fluorescent Protein, Veh = Vehicle, ns = not significant, Scale bars = 50µm.

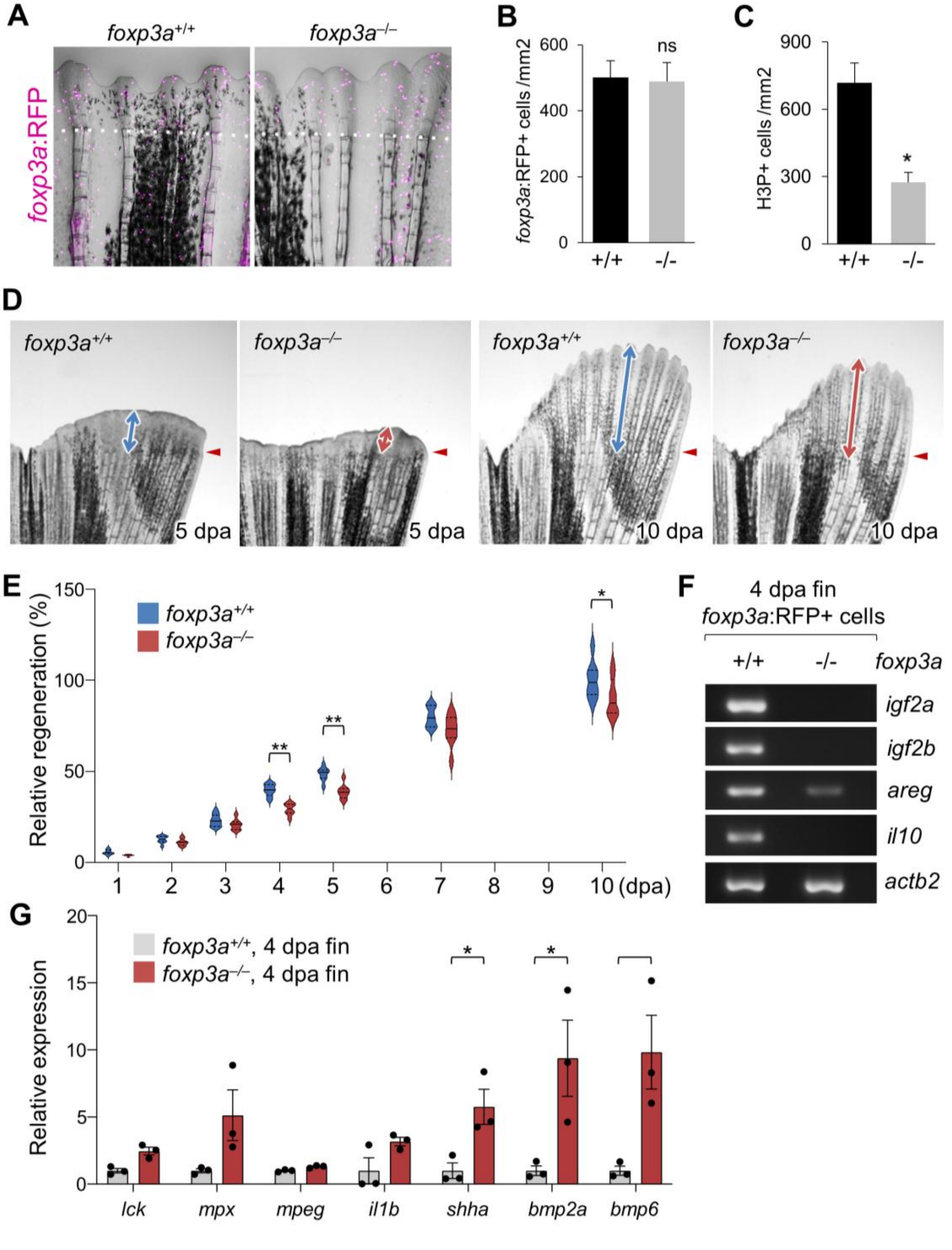

Supplementary Figure S3. Analysis of caudal fin regeneration in *foxp3a*$^{-/-}$ zebrafish. (A) Spatio-temporal distribution of *foxp3a:RFP*+ Tregs in the distal part of 4 dpa regenerating caudal fins of wild-type and *foxp3a*$^{-/-}$ zebrafish. Dotted lines show the plane of amputation. (B) Quantification of *foxp3a:*RFP+ cells in A (mean ± SEM, n = 8, Mann–Whitney *U* test). (C) Quantification of H3P+ cells in 4 dpa fin blastema of wild-type and

foxp3a$^{-/-}$ fish (mean ± SEM, n = 10, Mann–Whitney *U* test). (D) Brightfield microscopic images of caudal fins show the rate of fin regeneration after 4 and 10 dpa in wild-type and *foxp3a*$^{-/-}$ fish. (E) Rate of fin regeneration length was quantified in the wild-type fish against the *foxp3a*$^{-/-}$ fish as indicated in D (mean ± SEM, n = 8-9, Student's T-test). The average length of wild-type 10 dpa was considered as 100% length of fin regeneration. (F) RT-PCR analysis of growth factors and *il10* expression from purified *foxp3a:RFP*+ cells of wild-type and *foxp3a*$^{-/-}$ 4 dpa fin tissues. (G) qRT-PCR analysis of immune cell markers and inflammatory cytokines in 4 dpa fin blastema from wild- type and *foxp3a*$^{-/-}$ fish (mean ± SEM, n = 5, Student's T-test). Gene expression is shown relative to the levels in wild-type fish. *P < 0.01; **P < 0.001; ns = not significant, Scale Bar = 200 µm.

Supplementary Table S1. Primers used in qRT-PCR and RT-PCR analysis.

Gene	Forward primer sequence (5'–3')	Reverse primer sequence (5'–3')	Experiments
areg	ATCCTCATCATGGTCTGCGT	CACAGTCTTACATTCTCCTTTCACAC	qRT-PCR
areg	ACATACCTGCACGGCCTGAG	TCAGGAAAGCCGCCTGGAAG	semi qRT-PCR
bcl2a	TGGATCGAGGAAAATGGAGGT	AGCCGAGCACTTTTGTTAGG	qRT-PCR
bcl2l10	AGAAAAACAGGACTGGCTGG	ATGGAGGACTCCTGGTTCAG	qRT-PCR
bclXL	TGGATCCAAAGCCAAGGAGGA	GAAGCTTTCTTGCGATTTCCTGC	qRT-PCR
bmp2a	CAGAGCAAACACGATACGAAGT	CTCGTACTGGCATCTCCGAGAA	qRT-PCR
bmp6	ACGCTGACATGGTCATGAGT	AGTAGGAATGTCTCGTTCCGGA	qRT-PCR
fgf20a	CAAGGGCGAACTGTACGGAT	CCCCATCTCTGGACGTCCCA	qRT-PCR
foxp3a	ACCTCACCAAAACCAATGGC	TCTGATTGCGAGATGAGCCG	qRT-PCR
ifng1-1	GGGTTGGAAAATCTGTGTTTGT	GGAAAATGTCTTCATAGATGCTCA	qRT-PCR
igf1	ACAAGTTCATTTTTGCTGGGCT	TAGAGATCGTGGAGATTTGCCT	semi qRT-PCR
igf2a	TGCAGGTCTTCCCAGTGTCA	TCTGAGCAGCTTTTTGTTGCCA	qRT-PCR
igf2a	TGAAGTCGGAGCGAGATGTT	AGTGCCTCTACTGACCAAGAT	semi qRT-PCR
igf2b	AGCTGGTGGACGCTCTACA	GAGAACGTCGACTGTTTGACC	qRT-PCR
igf2b	ATCGCTGCAGGTCATTCCAG	ACTCTGTGCAAACGATCCTGT	semi qRT-PCR
il1b	CGCTCCACATCTCGTACTCAA	AACAGCAGCTGGTCGTATCC	qRT-PCR
il4	CCTGACATATATGAGACAGGACACTAC	TTACCCTTCAAAGCCATTCC	qRT-PCR
il6	AAGGGGTCAGGATCAGCAC	GCTGTAGATTCGCGTTAGACATC	qRT-PCR
il10	TGGAGACCATTCTGCCAACAG	TCCAACCCAGCAACATCCTATT	semi qRT-PCR
il10	CTTTGCGACTGTGCTCAGAG	TGGTTCCAAGTCATCGTTGGAC	qRT-PCR
il21	GGCTGACGAATTCAAACAAGA	AGAGCAGACGCAACACAGC	qRT-PCR
marco	GATACGACTTCCAGCACAGC	AACCCAAGCCTCCAGTGTAAA	qRT-PCR
mcl1a	CGAGTGGTCGACAATATTCTCGT	TCTGATGAAGTCCAGAGAGGC	qRT-PCR
mcl1b	TCTACTTACAGAGCAGCGGGA	TTCTCACAGCTGCCTCTGTA	qRT-PCR
nos2b	AACGGCATCATGAACTGTTG	TACATTGTAGTCCTCCATGCAAA	qRT-PCR
nr4a1	TTCTTGTCAGCATTACGGGGT	TGAGTCGATATGGGCAGTAACG	semi qRT-PCR
nrg1	AAAGGCCCTCAACACCCTCA	GTGGACGGATGTGTTGGTGA	semi qRT-PCR
nrp1a	ACACTTTGACCTGGAAGACCGA	AAATCTCCAGGCGGTCGTATC	semi qRT-PCR
ntf3	TACCTTCATGTCGGCTCTGC	CATTGGAGGTCGGTCGCTTA	semi qRT-PCR
raldh2	CTCTTCCACAGGGCCTGTAG	CTTCTTGGATCAGCTTGCCT	qRT-PCR
shha	AAGCCCACATTCATTGCTCT	CCTTCTGTCCTCCGTCCTG	qRT-PCR
tbx21	CGAGCACCCATGTACTCTTT	GCCAGAACCACATCCACGTA	qRT-PCR
tnfa	AGGCAATTTCACTTCCAAGG	AGGTCTTTGATTCAGAGTTGTATCC	qRT-PCR
wnt8a	GGAAAGCGCACTGCAGTTAT	CCGATCTTGGAGTCGTCACA	qRT-PCR
actb2	TGACTGACTACCTCATGAAGATCC	TCCAGACAGAGTATTTACGCTCAG	semi qRT-PCR
actb2	GCCTGACGGACAGGTCAT	ACCGCAAGATTCCATACCC	qRT-PCR
